# Narrative Review of Free Radicals and Reactive Oxygen Species in Otitis Media

**DOI:** 10.3390/antiox14010003

**Published:** 2024-12-24

**Authors:** Jeongmin Lee, Seok Min Hong, Yong Sung Choi, Jinseok Lee, Joon Hyung Yeo, Sung Soo Kim, Jae Min Lee, Dong Keon Yon, Seung Geun Yeo

**Affiliations:** 1 Department of Medicine, College of Medicine, Kyung Hee University Medical Center, Seoul 02447, Republic of Korea; sallyljm@khu.ac.kr; 2 Department of Otorhinolaryngology—Head and Neck Surgery, College of Medicine, Kyung Hee University Medical Center, Kyung Hee University, Seoul 02447, Republic of Korea; thecell20@gmail.com (S.M.H.); sunjaesa@hanmail.net (J.M.L.); 3 Department of Pediatrics, Kyung Hee University School of Medicine, Kyung Hee University Medical Center, Seoul 02447, Republic of Korea; feelhope@khu.ac.kr; 4 Department of Biomedical Engineering, Kyung Hee University, Seoul 02447, Republic of Korea; gonasago@khu.ac.kr; 5 Public Health Center, Danyang-gun 27010, Chungcheongbuk-do, Republic of Korea; joonhyungyeo@gmail.com; 6 Department of Biochemistry and Molecular Biology, College of Medicine, Kyung Hee University, Seoul 02447, Republic of Korea; sgskim@khu.ac.kr; 7 Center for Digital Health, Medical Science Research Institute, Kyung Hee University School of Medicine, Kyung Hee University Medical Center, Seoul 02447, Republic of Korea; 8 Department of Convergence Medicine, College of Medicine, Kyung Hee University, Seoul 02447, Republic of Korea

**Keywords:** free radicals, reactive oxygen species, otitis media

## Abstract

Many studies have evaluated the roles of free radicals and reactive oxygen species (ROS) in various diseases. To date, however, no systematic review has specifically investigated the involvement of free radicals and ROS in acute otitis media (OM), OM with effusion, and chronic OM. The present study therefore assessed the roles of free radicals and ROS in OM. SCOPUS, PubMed, Cochrane Library, EMBASE, and Google Scholar were comprehensively searched to identify studies assessing the roles of free radicals and ROS in OM. Relevant studies were identified and their findings summarized. The literature search identified 21 studies. A review of their findings showed that the concentrations of hydrogen peroxide (H_2_O_2_), lipid hydroperoxide (LPO), and myeloperoxidase (MPO) were elevated in patients with acute OM; that the concentrations of H_2_O_2_, LPO, MPO, xanthine oxidase (XO), superoxide dismutase (SOD), glutathione peroxidase (GPX), malondialdehyde (MDA), hydroxyl radical (·OH), and nitrotyrosine were elevated in patients with OM with effusion; and that the levels of nitric oxide (NO), MPO, 4-hydroxynonenal (4-HNE), and malondialdehyde (MDA) were elevated in patients with chronic OM. Although some studies reported that SOD levels were increased in patients with OM with effusion, decreases in antioxidants such as SOD and catalase, as well as total antioxidant capacity, were observed across all types of OM. Although the levels of free radicals and ROS varied by type of OM, study design, control group selection, sample type, ROS and free radical markers, and antioxidant types, most studies showed increased ROS and decreased antioxidants in OM patients. These findings suggest that levels of free radicals and ROS are strongly associated with the pathophysiology of OM.

## 1. Introduction 

### 1.1. Otitis Media—Acute Otitis Media, Otitis Media with Effusion, and Chronic Otitis Media 

Otitis media, an inflammatory disease that occurs in the middle ear, has been classified based on its duration, with otitis media lasting for less than 3 weeks classified as acute, otitis media lasting for 3 weeks to 3 months classified as subacute, and otitis media persisting for more than 3 months classified as chronic. Acute otitis media involves all acute inflammatory phenomena occurring in the middle ear cavity. In most patients, acute otitis media heals without sequelae; in others, however, the inflammation may recur or persist, manifesting as recurrent otitis media or otitis media with effusion. An incomplete resolution of the inflammation in the middle ear cavity may result in progression to chronic otitis media. The factors underlying the progression of acute infections in the middle ear and mastoid to chronic inflammation remain unclear. The incidence of acute otitis media is low in newborns, rises sharply after 6 months, peaks around age 2 years, and remains common in children aged 4 to 7 years. Acute otitis media occurring before age 2 years has been associated with a high likelihood of repeated episodes of acute otitis media [1,2,3].

Acute inflammation in the middle ear can pathophysiologically lead to the transformation and hyperplasia of the middle ear mucosa. This hyperplasia, as well as the influx of various inflammatory cells into the mucosa, is usually reversible. Thus, the removal of the stimuli associated with otitis media can induce a dedifferentiation process in the mucosa, which returns to its normal state. However, if hyperplasia and hyperproliferation of the middle ear mucosa repeatedly induce pathological conditions, such as middle ear effusion, atelectasis, adhesion, tympanosclerosis, and middle ear cholesteatoma, and they become chronic, irreversible structural changes may occur within the tympanic cavity. Therefore, although most cases of acute otitis media heal without sequelae, recurrent or persistent inflammation may lead to recurrent otitis media or otitis media with effusion and progression to chronic otitis media [4,5].

Otitis media with effusion is defined as the accumulation of effusion in the middle ear cavity without symptoms or signs of acute inflammation. This condition is very common in children, as well as being the most frequent cause of hearing loss in infants and young children. The main causes of otitis media with effusion include dysfunction of the Eustachian tube, adenoid hypertrophy, allergies, rhinosinusitis, and upper respiratory infections. The lack of a proper diagnosis and treatment of otitis media with effusion in infants and young children can lead to speech disorders due to hearing loss, decreased speech skills, behavioral disorders, and learning disabilities. Most cases of otitis media with effusion improve spontaneously with observation and follow-up, but patients who do not improve spontaneously may require surgical treatment, such as ventilation tube insertion. The higher prevalence of otitis media with effusion in infants and young children than in adults is related to the immaturity of the Eustachian tube. Eustachian tubes in infants and young children are not fully developed, as well as being shorter and positioned more horizontally than in adults. Thus, Eustachian tubes in infants and young children are less effective at protecting the middle ear from contamination by nasopharyngeal bacteria and pressure changes [6,7,8].

Chronic suppurative otitis media is a persistent inflammatory disease of the middle ear or mastoid. It is generally thought to be a complication of acute otitis media, especially in infants and young children. Chronic suppurative otitis media may also result from various traumas or chronic perforation of the tympanic membrane which remains after ventilation tube insertion. Typical findings in these patients include tympanic membrane perforation, thickening of the middle ear mucosa, granulomatous middle ear mucosa, and mucosal polyps. Additionally, the tympanic membrane can thicken or atrophy, often accompanied by myringosclerosis and tympanosclerosis around the middle ear mucosa and ossicles. Chronic tympanic membrane perforation without active infection or ear discharge is frequently called chronic otitis media. Chronic otitis media can cause changes such as perforation, atelectasis, retraction, adhesion, myringosclerosis, thickening, and atrophy of the tympanic membrane, and is sometimes accompanied by cholesteatoma. Depending on the degree of inflammation and the duration of the disease, chronic otitis media can lead to changes in the surrounding tissue and hearing loss; if not properly treated, chronic otitis media can cause various intracranial and extracranial complications [9,10,11].

Important pathological findings in chronic otitis media with perforation include granulation tissue formation, bone changes, myringosclerosis, cholesterol granuloma, cholesteatoma, and fibrosis. These lesions vary depending on whether the chronic otitis media is active or inactive. Granulation tissue can be categorized into immature forms, which are rich in blood vessels and cellular components, and mature forms, which have fewer cellular components and are rich in fibrous tissue. Active chronic otitis media has been associated with a significant amount of granulation tissue containing many cellular and vascular components [12,13,14,15].

### 1.2. Free Radicals/Reactive Oxygen Species (ROS)

**Figure 1:**
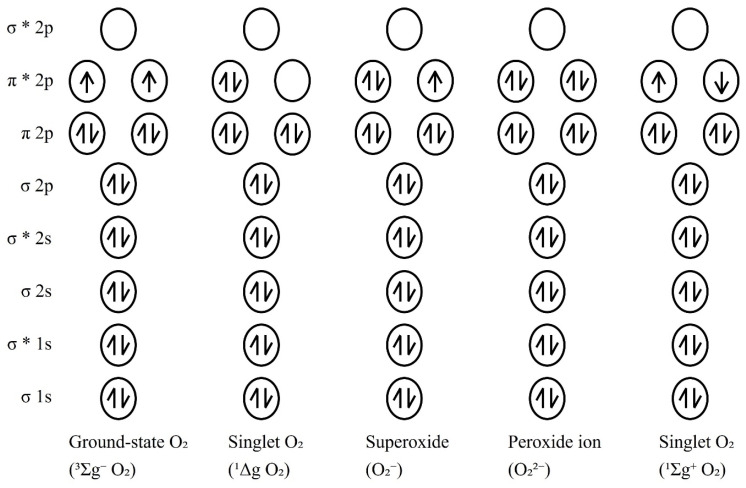
Bonding of diatomic oxygen molecules [19].

Free radicals, defined as independent molecules with one or more unpaired electrons, are highly prevalent in nature. Oxygen radicals are particularly important for living organisms, as they are the most abundant type of free radicals. Although most atmospheric oxygen is present as ground-state O_2_, oxygen can also form free radicals, including singlet O_2_ (^1^ΔgO_2_, ^1^ΣΔg⁺O_2_), superoxide (O_2_^−^), and peroxide ions (O_2_^2−^). Ground-state O_2_ that gains a single electron becomes the free radical O_2_^−^. If it gains an additional electron, it becomes O_2_^2−^, which is not a free radical but an ion, as it has two additional unpaired electrons. O_2_^2−^ can react with hydrogen ions to form hydrogen peroxide (H_2_O_2_). When a pair of electrons in ground-state O_2_ absorbs energy, the electrons become excited and transition to the singlet state. There are two singlet states, depending on the amount of energy absorbed: ^1^ΔgO_2_ (22.4 kcal) and ^1^ΣΔg⁺O_2_ (37.5 kcal) (Figure 1) [16,17,18].

Reactive oxygen species (ROS) are byproducts generated during the conversion of O_2_ into non-toxic water (H_2_O) as it receives four electrons. Specifically, O_2_ that gains one electron forms the superoxide anion (O_2_^−^), which is highly unstable. This unstable molecule is converted to H_2_O_2_ by the enzyme superoxide dismutase (SOD). H_2_O_2_ is more stable than the superoxide anion and is predominantly found within cells. The levels of H_2_O_2_ are tightly regulated and H_2_O_2_ is primarily detoxified by cellular antioxidant enzymes, such as catalase and peroxidase, which convert H_2_O_2_ into non-toxic H_2_O. However, if the amount of H_2_O_2_ produced is beyond the cell’s regulatory capacity, and the cell is unable to effectively eliminate it, H_2_O_2_ can gain an additional electron, forming the hydroxyl radical (·OH). This hydroxyl radical is highly toxic and is a major source of cellular damage. It can oxidize DNA, leading to the formation of DNA adducts, and can oxidize lipids, resulting in lipid peroxides, with both DNA adducts and lipid peroxides contributing to cellular toxicity. ROS, consisting of superoxide anions, H_2_O_2_, and hydroxyl radicals, are not merely byproducts of respiration, but can also function as secondary messengers in cellular signaling pathways. These signals are transiently generated in response to external stimuli through receptors on the cell membrane. Thus, while high concentrations of ROS in pathological conditions can act as toxic agents within cells, low concentrations of ROS generated temporarily in specific areas in response to external signals can serve as secondary messengers that regulate cell growth, apoptosis, and various cellular functions [20,21,22].

The human body has various antioxidant defense systems that maintain normal cellular membrane function and manage cellular metabolites, thereby counteracting injuries caused by ROS. Thus, ROS are continuously generated and eliminated, maintaining a balance between their production and removal, which is crucial for sustaining healthy cellular functions. These antioxidant defense systems can be broadly categorized as enzymatic and non-enzymatic. Important antioxidant enzymes in enzymatic defense systems include SOD, catalase (CAT), and glutathione peroxidase (GPX). Because ROS are generated in highly localized areas and have a short lifespan, these antioxidant enzymes are strategically distributed throughout the body to work synergistically to remove ROS. Moreover, these enzymes are produced endogenously, limiting the ability to supply them externally; therefore, research is focused on developing methods to maximize their endogenous production. Non-enzymatic defense systems include antioxidant substances such as vitamin C, vitamin E, β-carotene, uric acid, and bilirubin. These compounds react with ROS that are not specifically targeted by enzymatic actions, such as ·OH and singlet oxygen (^1^O_2_), to neutralize their toxicity. Unlike endogenous enzymes, such as SOD, CAT, and GPX, non-enzymatic antioxidants can be supplied externally, making them important subjects of research on modern anti-aging therapies (Table 1) [23,24,25,26].

**Figure 2:**
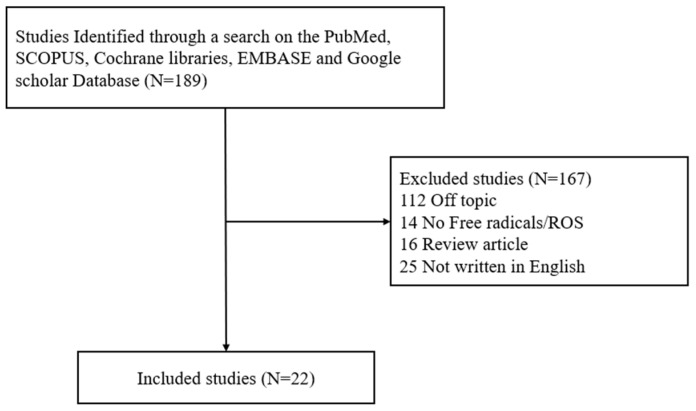
Flow diagram to identify studies included in the meta-analysis.

**Figure 3:**
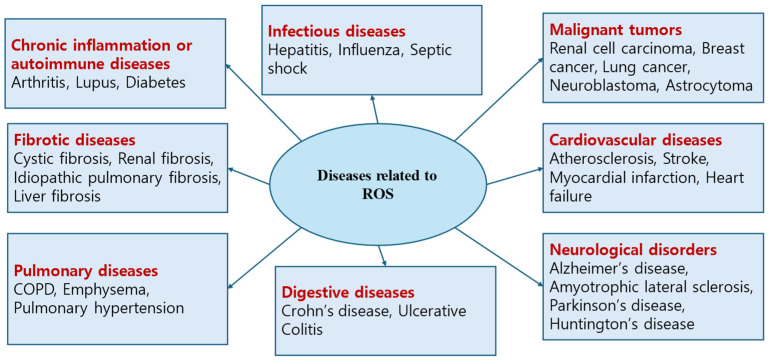
Diseases associated with ROS expression.

Although research on free radicals/ROS in various diseases is ongoing, there has been no established study on the role of free radicals/ROS in the occurrence and pathophysiology of otitis media. Therefore, studies examining free radicals/ROS in otitis media, published between January 1995 and July 2024, were retrieved from five electronic databases—PubMed, SCOPUS, Cochrane Library, EMBASE, and Google Scholar—by one of the authors (J.H.Y) based on the search terms “free radical/ROS” and “otitis media. The inclusion criteria were (1) prospective or retrospective studies on free radicals/ROS in otitis media; (2) studies on free radicals/ROS in normal middle ear; (3) human and animal studies were included. However, the exclusion criteria were (1) unpublished data; (2) review articles; (3) gray literature; (4) case reports; (5) duplicate studies; and (6) non-English publications. A total of 22 articles on free radicals/ROS in otitis media were identified and reviewed (Figure 2 and Figure 3).

## 2. Expression and Role of Free Radicals/ROS in Otitis Media

ROS associated with inflammatory diseases include the superoxide anion radical (O_2_^·−^), perhydroxyl radical (HO_2_·), hydrogen peroxide (H_2_O_2_), hydroxyl radical (HO·), singlet oxygen (^1^O_2_), hypochlorite ion (OCl-), and ozone (O_2_). Detoxifying enzymatic scavengers that inhibit or interfere with the function of these ROS include SOD, CAT, GPX, glutathione S-transferase, glutathione reductase, thioredoxin, and cytochrome c peroxidase, and detoxifying non-enzymatic scavengers include vitamin A, vitamin C, vitamin E, cysteine, coenzyme Q, uric acid, flavonoids, sulfhydryl groups, and thioether compounds. 

### 2.1. Free Radicals/ROS in Acute Otitis Media (AOM)

The free radicals and ROS involved in acute otitis media (AOM) include the superoxide anion radical (O_2_^·−^), H_2_O_2_, lipid hydroperoxide, and myeloperoxidase. Detoxifying enzymatic scavengers in AOM include SOD and CAT, and non-enzymatic scavengers in AOM include fisetin. A model of AOM was developed in 32 guinea pigs through the transtympanic injection of *Streptococcus pneumoniae* (Spn). H_2_O_2_ concentrations in middle ear fluid, an indicator of free radical activity, were measured at 6, 12, and 24 h. These concentrations were significantly higher in guinea pigs with AOM than in the control animals after 6 (0.74 versus 0.17), 12 (1.07 versus 0.47), and 24 (6.31 versus 1.08) hours (*p* < 0.05 each). Furthermore, H_2_O_2_ concentrations increased significantly over time in guinea pigs with AOM (*p* < 0.01). H_2_O_2_ is a central mediator in the generation of oxygen free radicals, leading to the formation of hydroxyl radicals (·OH) that induce cellular damage. These experimental results indicate that H_2_O_2_ is a significant contributor to free radical damage in the early phase of AOM [30]. 

Upon exposure to pathogens, neutrophils and macrophages generate H_2_O_2_. Because of the short half-life of free radicals, their direct measurement is challenging; thus, studies on free radicals often measure lipid hydroperoxide, a byproduct of free radical injury. Four studies have investigated lipid hydroperoxide in AOM. In one study, Spn was injected into 82 guinea pigs, which were sacrificed on days 5, 10, 20, and 30; their middle ear mucosae were collected and their lipid hydroperoxide contents were measured. Lipoperoxidation was highest on days 5 (*p* < 0.03) and 10 (*p* < 0.04), but decreased significantly by days 20 (*p* < 0.04) and 30 (*p* < 0.01). These findings suggested that lipoperoxidation plays a significant role in the pathogenesis of otitis media during the inflammatory phase following acute infection [31]. 

Another study found that lipid peroxidation (LPO) was affected by the use of antibiotics. In that study, Spn was injected into the middle ear of 78 guinea pigs to induce otitis media. The animals were then divided into groups receiving amoxicillin, Spn killed with amoxicillin, and a control group, and lipid hydroperoxide levels in the middle ear mucosa were measured on postoperative days 1 and 5 to assess free radical damage. On day 1, the mucosal lipid hydroperoxide levels were significantly higher in the two experimental groups than in the control group (*p* < 0.05). On day 5, lipid hydroperoxide expression was significantly higher in the mucosa infected with Spn than in the other two groups (*p* < 0.05). These findings indicate that free radical-induced lipid peroxidation occurs in the middle ear cavity during AOM, with similar results observed after treatment with antibiotics [32]. 

Another study evaluated the expression of LPO in the middle ear mucosa. AOM was induced in Hartley guinea pigs by Spn injection, and LPO concentrations were measured. The LPO levels were significantly higher in guinea pigs injected with Spn than in the control animals (*p* = 0.04), as well as being significantly higher than at baseline in the experimental group (*p* = 0.01). Additionally, submucosal edema, inflammatory cell infiltration, and hyperemia were observed in the experimental group [33]. In another study, LPO and malondialdehyde (MDA) levels were measured in the middle ear mucosa. Both LPO (*p* < 0.01) and MDA (*p* < 0.05) levels were found to be significantly higher in infected than in control mucosa [34], indicating that free radical damage occurs in the middle ear mucosa during pneumococcal otitis media. These findings suggested that free radicals generated by both the pathogen and neutrophils can damage the mucosal cell membrane, leading to thickening of the mucosa and functional impairment, which are critical aspects of AOM pathogenesis. Furthermore, these results suggested that treatment with antioxidants, such as vitamin C, vitamin E, beta-carotene, and scavenger enzymes (CAT, SOD, GPx), may be beneficial.

The effects of AOM on the expression of myeloperoxidase (MPO) were evaluated in C58BL/6 mice transtympanically inoculated with Spn. MPO is a major constituent of neutrophil granules and plays a crucial role in antimicrobial activity against intracellular microorganisms. However, elevated levels of MPO have been associated with various inflammatory diseases, including infections, ischemia, atherosclerosis, and acute myeloid leukemia (AML). Neutrophil MPO expression was found to be significantly higher in mice with Spn-induced AOM than in control mice (*p* < 0.05). Although MPO promoted the early-stage clearance of Spn, it also generated ROS and induced neutrophil apoptosis and necrosis, exacerbating tissue damage (*p* < 0.05). Furthermore, when treated with 4-aminobenzoic acid hydrazide (4-ABAH), an inhibitor of MPO, the experimental group exhibited significantly greater resistance to Spn than the control group (*p* < 0.05). These findings suggested that, although MPO is essential for protection against pathogens, its activity in the extracellular microenvironment can lead to various types of damage. In the context of AOM, MPO contributes to tissue damage by generating pro-inflammatory factors, such as lactate dehydrogenase (LDH), tumor necrosis factor-alpha (TNF-α), interleukin-1 beta (IL-1β), and ROS. The dual activity of MPO in Spn-induced AOM suggests that the appropriate regulation of MPO activity is crucial for balancing its protective and damaging effects, a balance that may play an important role in the treatment of Spn-induced AOM [35].

Free radicals can induce chemical modifications and cause subsequent damage to proteins, lipids, carbohydrates, and nucleotides. Intracellular antioxidant enzymes, including SOD, CAT, and GPX, protect tissues from free radical-induced damage. Several studies have investigated the correlations between the levels of expression of these antioxidants and improvements in AOM. SOD is a metalloprotein that converts superoxide anions into O_2_ and H_2_O_2_, thereby maintaining low intracellular concentrations of oxygen, a toxic metabolite. Additionally, SOD protects living tissues from the destructive effects of free radicals. A study of 20 Hartley guinea pigs with Spn-induced AOM found that the amount of submucosal edema was significantly greater in the experimental than in the control group. The SOD level was significantly higher in the normal than the in infected mucosa (1.77 ± 0.48 μg/mg protein vs. 1.02 ± 0.28 μg/mg protein, *p* < 0.05) [36]. Another study measured SOD levels in the tubal mucosa of four groups of 40 Sprague Dawley rats: a Non-AOM group, an AOM group, a recovery group, and a control group. There were no significant differences in the optical densities (213.5 ± 22.4 vs. 219.3 ± 18.7 vs. 223.5 ± 26.2) and surface areas (13.2 ± 0.8 mm^2^ vs. 14.8 ± 0.7 mm^2^ vs. 16.7 ± 0.4 mm^2^) of SOD of the Non-AOM, recovery, and control groups. Both the optical density (167.6 ± 19.3) and surface area (6.5 ± 0.9 mm^2^) of SOD, however, were significantly lower in the AOM than in the other three groups (*p* < 0.05 each) [37]. These results indicate that SOD, an intracellular antioxidant enzyme that protects cells from free radical damage, plays crucial roles in safeguarding the tubal mucosa from free radical injury during AOM and in preventing the progression of this condition.

CAT has shown patterns similar to those of SOD in AOM. A study of 20 mature guinea pigs with Spn-induced AOM found that the infected experimental group exhibited signs of inflammatory cell infiltration, congestion, and submucosal edema. Notably, the distribution of CAT was more restricted in the submucosal tissue of the experimental compared to the control animals. This staining pattern suggested that free radical damage due to inflammation in the colonic mucosa is also reflected in AOM, highlighting the significant role of inflammation-induced free radical damage in the pathogenesis of AOM [38]. 

In another study, AOM was induced in mice by injecting lipopolysaccharide (LPS) into the middle ear through the tympanic membrane, followed by the intragastric administration of fisetin for 10 days. Fisetin significantly reduced the LPS-induced increase in mucosal thickness, downregulated the release of pro-inflammatory cytokines, and inactivated the TLR4/NF-κB pathway. Fisetin also significantly reduced the production of ROS and inactivated the TXNIP/MAPKs signaling pathway. These findings suggested that fisetin improves AOM by inhibiting inflammation and ROS through the deactivation of the TLR4/NF-κB and TXNIP/MAPKs signaling pathways [39] (Table 2).

### 2.2. Free Radicals/ROS in Otitis Media with Effusion (OME)

Previous studies have evaluated ROS and free radicals associated with otitis media with effusion (OME), including O_2_^·−^, H_2_O_2_, ·OH, lipid hydroperoxide, myeloperoxidase, nitric oxide, xanthine oxidase, and malondialdehyde. To investigate the functions of these ROS and free radicals in OME, detoxifying enzymatic scavengers such as SOD and CAT were evaluated for study.

Lipid peroxidation (LPO) was measured in 94 middle ear fluid samples collected from 59 pediatric patients with OME. The average LPO concentration in these samples was 11.5 nmoles per million cells, significantly higher than the LPO concentrations in the control samples [40]. LPO levels were also measured in 35 patients with OME, with these concentrations being highest in mucoid effusions, followed by purulent and serous effusions (*p* < 0.05). Similarly, LPO per unit weight was highest in mucoid effusions, followed by purulent and serous effusions (*p* < 0.05). These differences may have been due to the distinct pathogenesis of the various types of effusions. Compared with mucoid and serous effusions, purulent effusions contain higher numbers of neutrophils and Streptococcus organisms, both of which generate free radical-like H_2_O_2_, thereby elevating LPO levels. In contrast, serous effusions contain relatively fewer neutrophils, likely resulting in lower LPO levels [41]. These findings suggest that inflammatory cells generate oxygen-derived free radicals, causing tissue injury in chronic OME, and that the elevated levels of oxidants in the middle ear play a significant role in the pathogenesis of chronic OME.

In vitro experiments were conducted using a primary cell culture-based ME model and a cell line obtained through the infection of ME epithelial cells from Mongolian gerbils with SV40. Xanthine oxidase was found to induce the generation of ROS, such as H_2_O_2_, O_2_·, and ·OH, thereby altering ionic transportation (short-circuit current of middle ear epithelium). Ionic transportation was maximal at 2 mM xanthine and 20 mU/mL xanthine oxidase, with this ROS-induced stimulation of ion transport being associated with an increase in cellular cAMP content (*p* < 0.05). CAT plays a role in inhibiting ROS by converting H_2_O_2_ to water, thereby reducing ion transport. Conversely, deferoxamine amplifies the effects of ROS through the Fenton reaction. Low concentrations of oxidants enhance prostaglandin synthesis and increase cellular cAMP levels, thereby increasing ion transport without deleterious cellular effects. However, high concentrations of oxidants can generate hydroxyl radicals through the Fenton reaction, leading to cellular toxicity and potential damage [42]. The opposing functions of these ROS are closely associated with middle ear pathology, underscoring the need to prevent excessive ROS from impairing mucociliary clearance in the middle ear.

A study involving 46 chinchillas injected with 7F *Streptococcus pneumoniae* found that the numbers of inflammatory cells, as well as their myeloperoxidase (MPO) concentrations, in middle ear fluid were significantly higher in the experimental than in the control group (*p* < 0.05). Additionally, the neutrophil MPO concentrations in peripheral blood 24 h post-inoculation were significantly higher in the experimental than in the control group (*p* < 0.001). In contrast, the neutrophil superoxide anion (O_2_^−^) levels in peripheral blood were similar in the two groups. The MPO levels in the experimental group were also found to increase over time. An in vitro experiment, in which nonviable pneumococci were introduced into neutrophils from middle ear fluid, found that both MPO and O_2_^−^ levels were lower at 24 h than in the animal experiments, although the levels became similar after 48 h. Conversely, the in vitro introduction of viable pneumococci resulted in lower MPO levels but similar O_2_^−^ levels compared with the animal experiments. These differences in MPO and O_2_^−^ levels suggest that different stimuli are required for their responses and may reflect intrinsic characteristics of these substances. Thus, oxidative metabolic products during the early phase of pneumococcal OME can accelerate middle ear damage [43]. 

Another study compared cell populations of CD4+ and CD8+ T cells, the CD4+/CD8+ ratio, the concentrations of the cytokines interleukin (IL)-2, IL-4, and IL-6, and the concentrations of immunoglobulin E (IgE) and nitric oxide (NO) in middle ear effusion (MEE) and the peripheral blood of 50 children with OME and 50 healthy children. The populations of CD4+ and CD8+ T cells, the CD4+/CD8+ ratio, and the concentrations of IgE and NO in peripheral blood were significantly higher in children with OME than in the control children (*p* < 0.01 each). In addition, the concentrations of IL-2, IL-6, IgE, and NO were significantly higher in MEE than in the peripheral blood of children with OME (*p* < 0.01 each). These findings suggest that the higher numbers of CD4+ and CD8+ T cells in peripheral blood, along with the elevated IL-2, IL-6, and NO levels in MEE, are associated with the pathogenesis of OME [44].

The expression of NO in patients with OME was found to be unaffected by patient age or by acute versus chronic phase; in that study, however, OME patients were not compared with a normal control group. In brief, middle ear fluid specimens were collected during tympanotomy or ventilation tube insertion from 90 patients with OME. These patients were grouped into those with acute (n = 50) and chronic (n = 40) OME and stratified by age into three groups: <16 years old (n = 13), 16–50 years old (n = 14), and >50 years old (n = 63). NO concentrations and SOD activity did not differ significantly by phase of OME or by patient age [45].

Oxygen free radicals (OFRs) are increasingly associated with the pathogenesis of various diseases and inflammatory conditions. OFRs can damage cells and tissues through the chemical modification of proteins, carbohydrates, nucleotides, and lipids. Under physiological conditions, OFRs are part of normal regulatory circuits and are neutralized by antioxidants. The pathogenesis of OME has been assessed using detoxifying enzymatic scavengers, such as SOD and CAT, to inhibit or interfere with the functions of ROS and free radicals. For example, 74 subjects were divided into three groups: 26 OME patients who underwent adenoidectomy with ventilation tube replacement (Group 1), 28 OME patients who underwent adenoidectomy without ventilation tube insertion (Group 2), and 20 healthy controls (Group 3). The concentrations of erythrocyte malondialdehyde (MDA) and glutathione peroxidase (GSH-Px) were found to be significantly higher in Group 1 than in Groups 2 and 3 (*p* < 0.05). SOD enzyme activity was lower in Group 1 than in Group 2 (*p* < 0.05), but higher in Group 1 than in Group 3 (*p* < 0.05). CAT enzyme activity was significantly lower in Group 1 than in Group 3 (*p* < 0.05). Middle ear inflammation increases the levels of free oxygen radicals in erythrocytes, with the damage caused by these free radicals being mitigated by antioxidant enzymes such as CAT, GSH-Px, and SOD. These findings suggested that a weakened antioxidant defense system contributes to the increased levels of free oxygen radicals associated with the pathogenesis of OME [46].

Another study compared various parameters, including erythrocyte total superoxide scavenger activity (TSSA), non-enzymatic superoxide scavenger activity (NSSA), SOD, CAT, xanthine oxidase (XO) activity, and MDA levels, in guinea pigs with and without OME. TSSA (*p* < 0.05), SOD (*p* < 0.05), XO (*p* < 0.05), and MDA (*p* < 0.01) levels were found to be significantly higher in the experimental OME group than in the control group, although there were no significant between-group differences in NSSA and CAT activities (*p* > 0.05 each) [47].

OFR production was found to be increased in experimental OME induced by histamine injection, indicating that OFRs play a crucial role in inducing OME-associated tissue damage. Unlike in AOM, SOD levels were higher in the middle ear effusion of subjects with OME than in the control group, suggesting that the pathogenesis of OME may differ from that of AOM. In addition, the defensive mechanisms in middle ear effusion may differ from those in blood or middle ear mucosa. Alternatively, the increase in SOD may be a compensatory response to suppress or neutralize the expression of ROS or free radicals following acute inflammation, thereby reducing the inflammatory responses within the middle ear (Table 3).

### 2.3. Free Radicals/ROS in AOM + OME

The Blood levels of MPO, GPx, CAT, NO, MDA, and SOD were measured in 107 children aged 2 to 13 years who were classified into three groups: 31 with AOM, 39 with chronic otitis media with effusion (COME), and 37 healthy controls. NO (*p* = 0.001), CAT (*p* = 0.044), and MPO (*p* = 0.04) levels were found to be significantly higher in the COME group than in both the AOM and control groups, with the CAT levels being lower in the AOM group than in both the COME and control groups. In contrast, the MDA (*p* = 0.365), GPx (*p* = 0.285), and SOD (*p* = 0.497) levels did not differ significantly across the three groups. Notably, CAT levels were low during the acute phase of otitis media, but increased as the condition progressed to the chronic phase. The oxidative stress marker that was highest in both the AOM and COME groups was NO, with MPO being particularly elevated in the COME group. These findings highlight the significant roles of oxidative stress and antioxidants in the pathogenesis of both AOM and COME [48] (Table 4).

### 2.4. Free Radicals/ROS in COM 

Although free radicals and oxidative stress are thought to play key roles in the pathogenesis of COM, their exact contributions remain inadequately explored. ROS and free radicals associated with COM include MPO, NO, and MDA, whereas SOD, CAT, total antioxidant capacity (TAC), and fisetin have been shown to inhibit or interfere with the functions of these in COM.

The involvement of free radicals and ROS in COM was investigated in 65 patients who underwent surgery for this condition, including 34 patients with tympanosclerotic plaques on the tympanic membrane, middle ear mucosa, or near the ossicular chain or mastoid bone (Group 1), and 31 patients without tympanosclerotic plaques (Group 2). Tissue NO and MDA levels in both middle ear mucosa (*p* = 0.001) and tympanic membrane (*p* = 0.01) specimens, as well as plasma CAT levels (*p* = 0.001), were significantly higher in Group 1 than in Group 2, whereas plasma SOD levels did not differ significantly between the two groups (*p* > 0.05). These results suggest that NO, free oxygen radicals, and CAT may play a role in the development of tympanosclerosis in patients with COM [49]. 

Despite the absence of a control group and differences in sample collection sites, conflicting findings have emerged in studies comparing COM patients. For example, a study involving 21 patients with COM with cholesteatoma, 40 patients with COM without cholesteatoma, and 30 healthy controls found that serum MPO, 4-HNE, MDA, and NO levels were significantly higher, whereas TAC levels were significantly lower, in the COM groups than in the control group (*p* < 0.001 each). Serum MPO, MDA, 4-HNE, and NO levels were also higher, and TAC levels lower, in COM patients with compared to those without cholesteatoma, although none of these differences was statistically significant (*p* > 0.05). These findings indicated that increased oxidative stress may play a crucial role in the pathogenesis of COM and subsequent tissue damage, suggesting that antioxidant therapy could be an alternative to surgery in patients with COM [50].

Other studies have reported, however, that NO is lower, not higher, in COM patients than in control subjects. Gaseous NO levels were found to be significantly higher in 11 patients with unilateral tympanic membrane perforation or Eustachian tube dysfunction than in 11 controls (9 ± 3 ppb vs. 4 ± 1 ppb, *p* = 0.04). The expression of endothelial NO synthase (eNOS) was found to be significantly lower in 45 COM patients than in controls (2.64 ± 0.86 mol/100,000 mol ACTB vs. 140.48 ± 92 mol/100,000 mol ACTB, *p* = 0.010), and the expression of neuronal NO synthase (nNOS) was significantly lower in 26 COM patients than in 15 control subjects (5.78 ± 1.13 × 10^−6^ ΔCt vs. 1.23 ± 0.32 × 10^−4^ ΔCt, *p* = 0.011). These findings suggested that NO is a natural and permanent component of the gas mixture in the human middle ear, with the presence of NOS enzymes in middle ear mucosa indicating ongoing NO production, which contributes to the pathogenesis of COM [51]. However, control group samples were taken from gas in the external ear canal opposite the lesion rather than from blood, middle ear mucosa, or inflamed tissue, which may have limited the comparability of these results with those of previous studies (Table 5).

### 2.5. Limitations

This study had several limitations. First, as a retrospective literature review, the subjects and methods were not standardized, leading to potentially different results across different studies. Second, there were no studies that applied experimental results in animal to humans showing similar outcomes in human otitis media. Third, although the physiological and pathological effects of free radicals and ROS can depend on their levels of secretion, no studies to date have accurately monitored these secretion levels. Fourth, it was challenging to compare different types of otitis media by measuring free radicals or ROS in specific samples, such as effusion fluid in patients with OME and granulation tissue in patients with COM. Fifth, no studies to date have monitored the effects of antioxidants on ROS over time. Sixth, in studies conducted on humans, only the final outcomes after the occurrence of otitis media were compared with the control group, with none of these studies comparing clinical manifestations or being based on the amount of ROS, at different disease stages, such as the initial, intermediate, and post-onset stages.

## 3. Conclusions

This study investigated the levels of expression and roles of free radicals and ROS in otitis media. The SCOPUS, PubMed, Cochrane Library, EMBASE, and Google Scholar databases were searched comprehensively for relevant studies, a search that yielded 21 articles. ROS associated with otitis media include O_2_^·−^, HO_2_·, H_2_O_2_, HO·, ^1^O_2_, OCl^−^, and O_2_. Detoxifying enzymatic scavengers include SOD, CAT, GPx, glutathione S-transferase, glutathione reductase, thioredoxin, and cytochrome c peroxidase, whereas non-enzymatic scavengers include vitamins A, C, and E, cysteine, coenzyme Q, uric acid, flavonoids, sulfhydryl groups, thioether compounds, and fisetin.

Although the expression and levels of free radicals and ROS were found to depend on the type of otitis media, study subjects, experimental methods, control group selection, and sampling of reactive species, most studies reported that free radicals and ROS were increased in otitis media, accompanied by a decrease in antioxidant levels. These findings suggest that free radicals and ROS are involved in the pathogenesis and progression of otitis media.

**Figure 4:**
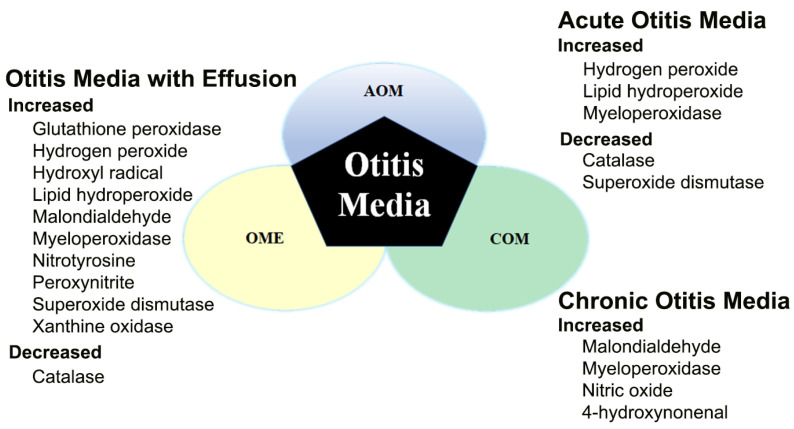
Patterns of expression of ROS and free radicals in AOM, OME, and COM.

The levels of free radicals and ROS were found to be elevated in AOM, OME, and COM. Antioxidants that inhibit these substances were found to improve the pathological state of otitis media and aid in its treatment. Agents and treatments that target free radicals and ROS may improve the pathogenesis of otitis media and contribute to its treatment without complications (Figure 4).

## Figures and Tables

**Table 1:** Types of reactive oxygen species and antioxidant defense mechanisms [27,28,29].

Type	Element Name	Enzyme	Reaction Catalysed
O_2_^·−^	Superoxide radical (or anion)	Superoxide dismutase	O_2_^·−^ + O_2_^·−^ + 2H^+^ → 2H_2_O_2_ + O_2_
HO_2_	Perhydroxyl radical	Catalase	2H_2_O_2_ → 2H_2_O + O_2_
H_2_O_2_	Hydrogen peroxide	Glutathione peroxidase	H_2_O_2_ + 2GSH → GSSG + 2H_2_O
HO·	Hydroxyl radical	Glutathione S-transferase	LOOH + 2GSH → GSSG + H_2_O + LOH
^1^O_2_	Singlet oxygen	Glutathione reductase	RX + GSH → RSG + HX *
OCL-	Hypochlorite	Thioredoxin	NADPH + GSSG ⇄ NADP^+^+ 2GSH
O_2_	Ozone	Cytochrome c peroxidase	Trx-(SH)_2_ + Protein-S_2_ ⇄ Trx-S_2_ + Protein-(SH)_2_
			Complex + 2cyt-c (Fe^2+^) → enzyme + 2cyt-c (Fe^2+^) + 2OH^−^
*Non-radicals*		*Nonenzymatic scavengers*	
H_2_O_2_	Hydrogen peroxide	Vitamin A	
HOCI	Hypochlorous acid	Vitamin C (ascorbic acid)	
		Vitamin E (-tocopherol)-carotene	
ONOO	Peroxynitrite	Cystein	
^1^O_2_	Singlet oxygen	Coenzyme Q	
		Uric acid	
		Flavonoids	
		Sulfhydryl group	
		Thioether compounds	

* R may be an aliphatic, aromatic, or heterocyclic group; X may be a sulfate, nitrite, or halide group.

**Table 2:** Free radicals/reactive oxygen species in acute otitis media.

Author/ Year/ Reference	Study Design	Species and/or Sample	Type of OM	Detection Method	Target Substance(s) Associated with Free Radicals	Results/Conclusions
Takodues T.G. et al., 1997 [30]	Animal study	32 guinea pigs injected transtympanically with *Streptococcus pneumoniae*	AOM	Histologic analysis, hydrogen peroxide assay	Hydrogen peroxide	Hydrogen peroxide levels increased with time after inoculation into infected ears (*p* < 0.01), and hydrogen peroxide levels were significantly higher in infected than in control middle ears at each time point < 0.05). Hydrogen peroxide, a component of oxygen free radical damage, was shown to be elevated in infected middle ear fluid in pneumococcal AOM. /This indicates that a neutrophil response to transtympanic injection was likely responsible for the generation of small amounts of H_2_O_2_ in these middle ears.
Haddad, J., Jr. et al., 1998 [31]	Animal study	82 guinea pigs injected transtympanically with *Streptococcus pneumoniae*	AOM	Histologic analysis, hydroperoxide assay	Lipid hydroperoxide	Free radical damage, evidenced by lipoperoxidation, which was previously found to contribute to the inflammatory changes associated with AOM. Lipoperoxidation was highest on days 5 (*p* < 0.03) and 10 (*p* < 0.04), significantly decreasing by days 20 (*p* < 0.04) and 30 (*p* < 0.01). /This indicates that lipoperoxidation may contribute to middle ear inflammation for a significant period of time after acute infection.
Takoudes T.G. et al., 2001 [32]	Animal study	78 guinea pigs that underwent bilateral middle ear inoculation with sterile saline (control), amoxicillin, *Streptococcus pneumoniae* killed with amoxicillin, and *S. pneumoniae*	AOM	Protein assays and histological evaluation	Lipid hydroperoxide	Mucosal lipid hydroperoxide significantly higher in the mucosa of both the antibiotic-killed *S. pneumoniae* group and the *S. pneumoniae*-infected group than in the control group on day 1 and significantly higher in the mucosa of the *S. pneumoniae*-infected group than in the mucosa of the antibiotic-killed and control groups on day 5. /This indicates that free radical damage to the middle ear mucosa may occur in otitis media, despite appropriate antibiotic therapy.
Takoudes T.G. et al., 1999 [33]	Animal study	21 Hartley guinea pigs injected with a suspension of type 3 *S. pneumoniae.*	AOM	Lipid peroxide assay, histologic evaluation	Lipid peroxide	Total LPO levels significantly higher in fluid from infected than control animals (*p* = 0.04). LPO concentration in the middle ear fluid, defined as total LPO divided by the weight of the fluid collected, was significantly higher (*p* = 0.01) in specimens from infected than control guinea pigs. Histologic examination confirmed leukocyte infiltration and mucosal edema that were consistent with mucosal damage. /Free radicals, which are likely to arise from both pathogens and neutrophils during infection, appear to attack mucosal cell membranes.
Dong Y. et al., 2021 [34]	Animal study, in vitro	Mouse model of AOM established by transbullar injection of *S. pneumoniae*	AOM	Western blotting, cell culture, confocal microscopy, fluorescence microscopy	NETs, TLR4, ROS	Otitis media caused mainly by *Streptococcus pneumoniae* (Spn) NETs present in the middle ear of animals with Spn-induced AOM. TLR4 contributed to the regulation of NET formation via autophagy activation and ROS production. TLR4 partly mediated NET formation in response to Spn in vitro and in vivo during AOM, producing NETs able to engulf and kill Spn both in vivo and in vitro. /TLR4 regulates ROS and autophagy to control the formation of NETs against Spn during the course of AOM.
Xiang Y. et al., 2017 [35]	Animal study	Model of AOM in C57BL/6 mice by direct bilateral transtympanic inoculation of *S. pneumoniae* into middle ears	AOM	MPO kinetic–colorimetric assay, flow cytometry, histopathologic analysis, lactate dehydrogenase assay, ELISA, immunofluorescence assay	MPO, ROS	MPO production by recruited neutrophils significantly higher in Spn-infected than in control mice, with neutrophils killing Spn in an MPO-dependent manner. MPO facilitated the generation of ROS, promoted Spn clearance at an early stage and exacerbated tissue damage. /MPO has dual roles in AOM, contributing to the removal of bacteria in the middle ear cavity, while also causing tissue damage partly associated with its ability to increase ROS generation.
Parks R.R. et al., 1995 [36]	Animal study	20 Hartley guinea pigs injected in the right ear with *S. pneumoniae* and in the left ear with sterile normal saline	AOM	Immunohistochemistry, Western blotting, tissue assay		SOD concentration significantly higher in normal (1.77 ± 0.48 μg/mg protein) than in infected (1.02 ± 0.28 μg/mg protein) mucosa (*p* < 0.05). /Infected ears showed a disproportionate increase in the ratio of submucosa to overlying epithelium, increasing the ratio of relatively SOD-poor to SOD-rich tissue.
Lee E.S. et al., 2000 [37]	Animal study	40 Sprague Dawley rats injected with *S. pneumoniae* and divided into three groups according to their tympanic cavity conditions: no-AOM, AOM, and recovery groups.	AOM	Immunohistochemistry, Western blot	SOD	Western blotting of samples from the Non-AOM, recovery, and control groups showed that optical density (213.5 ± 22.4 vs. 219.3 ± 18.7 vs. 223.5 ± 26.2) and surface area (13.2 ± 0.8.mm^2^ vs. 14.8 ± 0.7 mm^2^ vs. 16.7 ± 0.4 mm^2^) were similar, but that both optical density (167.6 ± 19.3) and surface area (6.5 ± 0.9 mm^2^) were markedly lower in the AOM group. /These findings suggest that superoxide dismutase may play a role in protecting tubal mucosa from free radical injury during AOM.
Parks R.R. et al., 1996 [38]	Animal study	20 mature guinea pigs injected with *S. pneumoniae.*	AOM	Immunohistochemistry, Western blotting, ELISA	Catalase	The submucosal tissues in the infected middle ears stained poorly for catalase, suggesting that their catalase contents were lower than those of the epithelium. This staining pattern was similar to that of SOD. This result was expected, as the two enzymes have complementary functions. /The antioxidant enzymes catalase, glutathione peroxidase, and superoxide dismutase protect tissues from the destructive effects of free radicals, with these enzymes having complementary functions.
Li et al., 2018 [39]	Animal study	60 male C57BL/6 mice injected with LPS into the middle ear via the tympanic membrane, followed by intragastric administration of fisetin.	AOM	H&E staining, ELISA, RT-qPCR, Western blotting, flow cytometry, immunohistochemistry	ROS, MDA, SOD	The concentrations of the pro-inflammatory cytokines, IL-1β, TNF-α, IL-6, and VEGF, were high in LPS-treated mice, with all of these concentrations being reduced by fisetin administration. This process was dependent on TLR4/NF-kB inactivation. LPS reduced serum SOD and MDA activities, reductions reversed by fisetin administration. /Fisetin can improve the symptoms of AOM by inhibiting inflammatory responses and reducing oxidative stress.

Abbreviations: AOM, acute otitis media; CAT, catalase; COM, chronic otitis media; ELISA, enzyme linked immunosorbent assay; FR, free radical; FOR, free oxygen radical; GSH, glutathione; GSH-Px, glutathione peroxidase; LDH, lactic dehydrogenase; LPO, lipid peroxidation, lipid hydroperoxide; MDA, malondialdehyde; ME, middle ear; MEE, middle ear effusion; MEF, middle ear fluid; MPO, myeloperoxidase; NET, neutrophil extracellular trap; NO, nitric oxide; NT, nitrotyrosine; OM, otitis media; OME, otitis media with effusion; OFR, oxygen free radical; PN, peroxynitrite; PGE2, prostaglandin E2; ROS, reactive oxygen species; SOD, superoxide dismutase; TLR, toll like receptor; TSSA, total superoxide scavenger enzyme activity; XO, xanthine oxidase.

**Table 3:** Free radicals/reactive oxygen species in otitis media with effusion.

Author/ Year/ Reference	Study Design	Species and/or Sample	Type of OM	Detection Method	Target Substance(s) Associated with Free Radicals	Results/Conclusions
Testa D. et al., 2012 [40]	Comparative study	59 children with a history of middle ear effusion unresponsive to repeated medical treatments who underwent myringotomy.	OME	Flow cytometry	Lipid peroxides, free radicals, glutathione	Lipid peroxide levels in all samples were high (mean 11.5 nmole/million cells), indicating a high level of oxidative stress and a high percentage of inflammatory cells. /Inflammatory cells in chronic OME generate high levels of oxygen-derived free radicals. The improvement induced by GSH treatment while applying ventilation tubes and after surgery can prevent this possible oxidative stress.
Takoudes T.G. et al., 1999 [41]	Comparative study	35 specimens of middle ear fluid from children with chronic otitis media classified as mucoid (n = 19), purulent (n = 10), or serous (n = 6).	OME	LPO assay	Lipid hydroperoxide	The significantly higher levels of total LPO in mucoid than in serous effusions and of LPO concentrations in purulent than in serous effusions may be explained by the pathogenesis of these effusions. Purulent effusions may contain both increased neutrophils and Streptococcus organisms, which produce FR-like hydrogen peroxide, increasing LPO levels. Serous effusions are likely to contain fewer neutrophils, resulting in a lower level of lipid peroxidation. /LPOs are present in fluid recovered from middle ears of pediatric patients with OM. The total amount of LPO (in nanomoles) is significantly higher in mucoid than in serous effusions, and the LPO concentration (nanomoles per microgram of fluid) is significantly higher in purulent than in serous effusions.
Parks R.R. et al., 1994 [42]	Animal study	20 Hartley guinea pigs injected with *Streptococcus pneumoniae*	Experimental Otitis Media	Histologic analysis, tissue assays; lipid hydroperoxide and thiobarbituric acid (TBA) assays	LPO and MDA	The concentrations of LPO (*p* < 0.01) and MDA (*p* < 0.05) were found to be significantly higher in infected mucosa than in saline-injected controls. /Free radicals play a role in the pathogenesis of otitis media.
Kawana M. et al., 1991 [43]	Animal study, in vitro	46 chinchillas injected with 7F *S. pneumoniae*	OME	Biochemical and colorimetric assays	MPO, Superoxide anion	In vitro stimulated production of MPO and O2^−^ was significantly lower from middle ear than from peripheral blood neutrophils 24 h after inoculation of nonviable pneumococci, although these levels were similar after 48 h. 24 h after inoculation of viable pneumococci, middle ear neutrophils stimulated in vitro produced less MPO but the same amount of O^2−^ as did blood neutrophils. /Oxidative metabolic products are released from phagocytic cells into the MEF during OM.
Fan W. et al., 2019 [44]	Comparative study	50 children with OME, 50 healthy children as controls	OME	Flow cytometry and ELISA	Immunoglobulin E, NO, cytokines, T lymphocytes	The percentages of peripheral blood CD4+ and CD8+, the CD4+/CD8+ ratio, and IgE and NO levels were significantly higher in children with than without OME (*p* < 0.01 each). In children with OME, the levels of IL-2, IL-6, IgE, and NO were significantly higher in the MEE than in peripheral blood (*p* < 0.01 each). /The levels of CD4+ and CD8+ T lymphocytes in peripheral blood and of IL-2, IL-6, IgE, and NO in MEE are increased in children with OME.
Hisamatsu K. et al., 2005 [45]	Comparative study	90 patients with OME, including 50 with acute and 40 with chronic OME. Patients were also classified by age: <16 years (group A, n = 13), 16–50 years (group B, n = 14), and >50 years (group C, n = 63).	OME	ELISA and colorimetric assays	Nitrotyrosine, NO, SOD, LDH	NT concentration was significantly higher in group A than in group C (*p* < 0.05), and significantly higher in patients with acute than chronic OME (*p* < 0.05). NO concentration did not differ significantly among patient groups. SOD activity correlated significantly with NT and NO concentrations and LDH activity (*p* < 0.05 each). LDH activity was significantly greater in group A than in group C (*p* < 0.05). /The NO–superoxide system is involved in the pathogenesis of OME, providing evidence for protein and/or cell injury in the middle ear.
Yariktas M. et al., 2004 [46]	Comparative study	74 subjects, including 26 OME patients who underwent adenoidectomy with ventilation tube replacement (Group 1), 28 age-matched OME patients who underwent adenoidectomy without ventilation tube insertion (Group 2), and 20 healthy controls (Group 3).	OME	Blood parameters	MDA, SOD, CAT, GSH-Px enzymes	Erythrocyte MDA level and GSH-Px enzyme activity in blood samples were significantly higher in Group 1 than Groups 2 and 3 (*p* < 0.05 each). SOD enzyme activity in blood samples was significantly lower in Group 1 than in Group 2 (*p* < 0.05) and significantly higher in Group 1 than in Group 3 (*p* < 0.05). CAT enzyme activity was significantly higher in Group 1 than Group 3 (*p* < 0.05). /Inflammation of the middle ear increases the levels of FORs in erythrocytes. FOR levels are normally maintained at steady state by antioxidant enzymes. Weakening of the antioxidant defense system increases FORs, contributing to OME.
Aktan B. et al., 2003 [47]	Animal study	12 guinea pigs, six injected with histamine solution and six injected with normal saline.	OME	Biochemical parameters	SOD, CAT, XO, MDA, TSSA	The MDA level, TSSA, SOD, and XO activities in the erythrocytes of the experimental OME group were significantly higher than those of the control group (*p* < 0.05, for the first three parameters; *p* < 0.01, for the last one). The TSSA and CAT activities in erythrocytes of the experimental OME group did not differ significantly from those of the control group. /This suggests that OFRs may play an important role in cell and tissue damage due to OME.

Abbreviations: CAT, catalase; ELISA, enzyme linked immunosorbent assay; FR, free radical; FOR, free oxygen radical; GSH, glutathione; GSH-Px, glutathione peroxidase; LDH, lactic dehydrogenase; LPO, lipid peroxidation, lipid hydroperoxide; MDA, malondialdehyde; ME, middle ear; MEE, middle ear effusion; MEF, middle ear fluid; MPO, myeloperoxidase; NO, nitric oxide; NT, nitrotyrosine; OM, otitis media; OME, otitis media with effusion; OFR, oxygen free radical; SOD, superoxide dismutase; TSSA, total superoxide scavenger enzyme activity; XO, xanthine oxidase.

**Table 4:** Free radicals/reactive oxygen species in acute otitis media and otitis media with effusion.

Author/ Year/ Reference	Study Design	Species and/or Sample	Type of OM	Detection Method	Target Substance(s) Associated with Free Radicals	Results/Conclusions
Sagiroglu S. et al., 2018 [48]	Comparative study	31 AOM, 39 COME patients, and 37 control subjects.	AOM, OME	Blood parameters	MPO, GPx, CAT, NO, MDA, SOD	MPO (*p* = 0.040), NO (*p* = 0.001), and CAT (*p* = 0.044) were significantly higher in the AOM and COME groups than in the control groups. CAT was low during the acute phase and high during the chronic phase of OM. MPO and CAT distributions were significantly greater in the COME group. CAT was lower in the AOM than in the COME and control groups. MDA, GPx, and SOD distributions were not significantly different in the three groups. /Serum levels of oxidative stress markers and antioxidants play an active role in the pathogenesis of COME and AOM.

Abbreviations: AOM, acute otitis media; CAT, catalase; COME, chronic otitis media with effusion; GPx, glutathione peroxidase; MDA, malondialdehyde; MPO, myeloperoxidase; NO, nitric oxide; SOD, superoxide dismutase.

**Table 5:** Free radicals/reactive oxygen species in COM.

Author/ Year/ Reference	Study Design	Species and/or Sample	Type of OM	Detection Method	Target Substance(s) Associated with Free Radicals	Results/Conclusions
Karhdag T. et al., 2004 [49]	Comparative study	65 patients with COM, including 34 with tympanosclerotic plaques on the tympanic membrane, middle ear mucosa, ossicular chain, or mastoid bone, and 31 without these plaques, along with 30 controls.	COM	Venous blood, middle ear mucosa, and tympanic membrane	NO, SOD, Malondialdehyde, Catalase	NO and MDA levels in middle ear mucosa (*p* = 0.001) and tympanic membrane (*p* = 0.01), as well as plasma MDA and CAT levels (*p* = 0.001 each), were significantly higher in COM patients with than those without tympanosclerotic plaques. SOD levels were similar in the two groups (*p* > 0.05). /Increased oxidative stress seems to be associated with decreased antioxidant levels in patients with COM, suggesting a role for increased oxidative stress in the pathogenesis of COM.
Garca M.F. et al., 2013 [50]	Comparative study	61 patients, 21 with and 40 without cholesteatoma.	COM	Spectrophotometer, chromatographic spectrometer, ELISA	MPO, NO, MDA, 4-HNE	Serum MPO activity and MDA, 4-HNE, and NO levels were significantly higher, and TAC levels significantly lower, in COM patients than in controls (*p* < 0.001 each). Serum MPO activity and MDA, 4-HNE, and NO levels were higher in COM patients with than without cholesteatoma, but between-group differences were not statistically significant (*p* > 0.05). /Increased oxidative stress may play a role in the pathogenesis of COM.
Johanna Westerberg J. et al. 2022 [51]	Case–control study	Gaseous NO from 11 patients with unilateral perforations. Middle ear mucosa from 48 patients with COM and 26 with cholesteatoma.	COM	Chemiluminescence	NOS	The gaseous NO concentration was higher in ears with a unilateral tympanic membrane perforation or grommet than in controls (*p* = 0.04). eNOS levels were lower in pooled samples from COM patients than controls (*p* = 0.010), and nNOS levels were lower in COM patients with cholesteatoma than in controls (*p* = 0.011). /NOS enzymes in the middle ear mucosa are indicative of ongoing NO production. Reduced NOS in ears with cholesteatoma and in pooled samples from ears with COM suggests a role for locally produced NO in middle ear disease.

Abbreviations: COM, chronic otitis media; ELISA, enzyme linked immunosorbent assay; MDA, malondialdehyde; MPO, myeloperoxidase; NO, nitric oxide; SOD, superoxide dismutase; TAC, total antioxidant capacity; 4-HNE, 4-hydroxynonenal.
